# Differential hypoglycaemic, anorectic, autonomic and emetic effects of the glucagon-like peptide receptor agonist, exendin-4, in the conscious telemetered ferret

**DOI:** 10.1186/s12967-014-0327-6

**Published:** 2014-12-10

**Authors:** Zengbing Lu, Nathalie Percie Du Sert, Sze Wa Chan, Chi-Kong Yeung, Ge Lin, David TW Yew, Paul LR Andrews, John A Rudd

**Affiliations:** School of Biomedical Sciences, Faculty of Medicine, The Chinese University of Hong Kong, Shatin, New Territories, Hong Kong, SAR China; Division of Biomedical Sciences, St George’s University of London, London, UK

**Keywords:** Blood glucose, Exendin-4, Exendin (9–39), Ferret, Gastric myoelectric activity, Glucagon-like peptide 1, Heart rate variability, Nausea, Vomiting

## Abstract

**Background:**

Rodents are incapable of emesis and consequently the emetic potential of glucagon-like peptide-1 receptor (GLP-1R) agonists in studies designed to assess a potential blood glucose lowering action of the compound was missed. Therefore, we investigated if the ferret, a carnivore with demonstrated translation capability in emesis research, would identify the emetic potential of the GLP-1R agonist, exendin-4, and any associated effects on gastric motor function, appetite and cardiovascular homeostasis.

**Methods:**

The biological activity of the GLP-1R ligands was investigated *in vivo* using a glucose tolerance test in pentobarbitone-anesthetised ferrets and *in vitro* using organ bath studies. Radiotelemetry was used to investigate the effect of exendin-4 on gastric myoelectric activity (GMA) and cardiovascular function in conscious ferrets; behaviour was also simultaneously assessed. Western blot was used to characterize GLP-1R distribution in the gastrointestinal and brain tissues.

**Results:**

In anesthetised ferrets, exendin-4 (30 nmol/kg, s.c.) reduced experimentally elevated blood glucose levels by 36.3%, whereas the GLP-1R antagonist, exendin (9–39) (300 nmol/kg, s.c.) antagonised the effect and increased AUC_0–120_ by 31.0% when injected alone (*P* < 0.05). In animals with radiotelemetry devices, exendin-4 (100 nmol/kg, s.c.) induced emesis in 1/9 ferrets, but inhibited food intake and decreased heart rate variability (HRV) in all animals (*P* < 0.05). In the animals not exhibiting emesis, there was no effect on GMA, mean arterial blood pressure, heart rate, or core body temperature. In the ferret exhibiting emesis, there was a shift in the GMA towards bradygastria with a decrease in power, and a concomitant decrease in HRV. Western blot revealed GLP-1R throughout the gastrointestinal tract but exendin-4 (up to 300 nM) and exendin (9–39), failed to contract or relax isolated ferret gut tissues. GLP-1R were found in all major brain regions and the levels were comparable those in the vagus nerve.

**Conclusions:**

Peripherally administered exendin-4 reduced blood glucose and inhibited feeding with a low emetic potential similar to that in humans (11% vs 12.8%). A disrupted GMA only occurred in the animal exhibiting emesis raising the possibility that disruption of the GMA may influence the probability of emesis occurring in response to treatment with GLP-1R agonists.

## Background

Glucagon-like peptide-1 (GLP-1) is a hormone secreted by the small intestine in response to nutrient ingestion [1]. It exerts its biological actions via G-protein-coupled receptors, which are members of the glucagon receptor superfamily, including glucagon, glucagon-like peptide-2, glucose-dependent insulinotropic peptide, growth hormone-releasing hormone, and secretin [2]. Activation of GLP-1 receptors reduces blood glucose levels by stimulating glucose-induced insulin secretion and suppressing glucagon production [3,4]. In addition, there is an inhibition of gastric emptying [5] and small bowel motility [6], with studies reporting a decrease of appetite and a reduction of food intake [7,8].

GLP-1 analogues have been investigated for the treatment of type-2 diabetes and obesity [9]. However, the use of such agents was commonly associated with nausea and emesis (vomiting) thus limiting their clinical development [10,11] as illustrated by the withdrawal of a GLP-1 analog in a phase III clinical trial because of the high incidence of nausea and vomiting [12]. The mechanisms and pathways by which GLP-1 receptors induce emesis are not known and this is partially because most preclinical research on GLP-1 used species incapable of emesis, such as rats and mice [13-15]. In these species, GLP-1 clearly reduces plasma glucose levels at doses that are known to delay gastric emptying and cause hypertension [16,17]. However, studies using the house musk shrew (*Suncus murinus*), an insectivore capable of emesis, have shown that the GLP-1 agonist, exendin-4 can lower blood glucose levels, elevate plasma insulin, induce emesis, and contract the isolated ileum via a mechanism involving the enteric nervous system [18-20].

To gain an insight into the mechanisms involved in the emetic dose-limiting toxicity of GLP-1 receptor agonists, we undertook a series of studies in the ferret, a species with a well characterised emetic response, sensitivity to a diverse range of emetic agents and demonstrated translation potential in efficacy of anti-emetic drugs [21,22]. Initially, the presence of GLP-1 receptors was established in the brain and gut and the effect of exendin-4 on isolated gut regions investigated. The biological activity of the GLP-1 receptor agonist exendin-4, and the GLP-1 receptor antagonist, exendin (9–39), was demonstrated *in vivo* using a glucose tolerance test [23]. As gastric dysrhythmia has been associated with nausea and emesis induced by a variety of treatments [24], we used telemetry to investigate the effect of exendin-4 on the gastric myoelectric activity (GMA) in conscious ferrets [25] and combined this with measurement of heart rate variability, an index of autonomic nervous system activity frequently monitored in human studies of nausea and vomiting [26,27], thus permitting further assessment of the potential for ferret data to translate to humans.

## Methods

### Animals

Twenty-eight castrated male fitch ferrets (1.51 ± 0.05 kg) were obtained from Southland Ferrets (Invercargill, New Zealand) and housed in a temperature-controlled room at 24 ± 1°C under artificial lighting, with lights on between 06:00 to 18:00 h. The relative humidity was maintained at 50 ± 5 %. Water and food (TriPro super premium chicken meal formula dog food, American Nutrition, USA) were given *ad libitum* unless otherwise stated. All experiments were conducted under licence from the Government of Hong Kong SAR and the Animal Experimentation Ethics Committee, The Chinese University of Hong Kong.

### Western blot

Animals (n = 3) were fasted overnight and then killed using pentobarbitone (80 mg/kg, i.p.) (Dorminal®, Alfasan, Woerden, Holland). The whole gastrointestinal tract was removed and immediately washed in freshly prepared Krebs solution (composition in mM: NaCl 118, KCl 4.7, KH_2_PO_4_ 1.2, MgSO_4_•7H_2_O 1.2, CaCl_2_•2H_2_O 2.5, NaHCO_3_ 25 and glucose 10) (Merck, Germany) and gassed with 95% O_2_ and 5% CO_2_ at room temperature; cervical vagi and brains were also removed. Segments of the gastrointestinal tract, the cervical vagi, and selected brain areas [28] were lysed in sodium dodecyl sulphate (SDS) lysis buffer containing protease inhibitor cocktail tablets (Complete Mini, Roche). The protein concentrations of each extract was measured using a Bio-Rad Protein Assay Kit (Bio-Rad Laboratories, Hercules, CA, USA) according to manufacturer’s instructions, and 12.5 μg of brain tissues or 25 μg of vagus or gut tissues were added to 10% polyacrylamide gels. Gels were then transferred to nitrocellulose membranes, blocked by incubating them with 5% bovine serum albumin in washing buffer for 1 h at room temperature, and then incubated overnight at 4°C with primary antibodies directed against the GLP-1 receptors (ab13181, 1:1000, Abcam, Inc., Cambridge, MA). This antibody reacts with mouse, rat, dog and human GLP1 R but no data are available on its specificity for the ferret receptor. Rat brain tissue was used as a positive control for the antibody (data not shown). Membranes were washed 3 times with 5 min intervals, incubated with anti-rabbit IgG horseradish peroxidise conjugate (1:2000, Thermo Scientific, Rockford, USA) for 1 h, and washed again (4 × 5 min) before incubating with a chemiluminescence detection reagent for 5 min. The protein was visualised by exposing the membrane to a ChemDoc XRS detection system (Bio-Rad, Milan, Italy). GAPDH served as an internal control, and it was similarly detected using a horseradish peroxidase conjugated mouse anti-GAPDH as the primary antibody. Band intensity was analysed and GLP-1 receptor expression was calculated as the ratio of the GLP-1 receptor band intensity to GAPDH band intensity in the same blot [29].

### Organ bath studies

One centimetre segments of circular antrum, longitudinal duodenum, jejunum, ileum and colon were dissected from animals killed for the Western blot study (see above). The segments with intact mucosa were mounted longitudinally under 0.5 g resting tension in a 10 ml organ bath containing Krebs solution and gassed with 95% O_2_ and 5% CO_2_ at 37°C [30]. The isometric contractions of tissues were recorded using Grass transducers via a MacLab® system (ADInstruments Pty Ltd., New South Wales, Australia ) connected to a Power Macintosh G3 computer (Apple Computer, Inc., California, U.S.A.). Analytical software (Chart, version 3.5 s/s MacLab®, New South Wales, Australia) was used to display and analyse the amplitude and frequency of the contractions. The contractile responses were measured by the change of tension (g) before and after the addition of drugs. The frequency of contractions was measured over a period of 1 min before and after drug administration. After 30 min, KCl (120 mM) was added to provide a reference contractile response followed by washout.

After 60 min of equilibration, increasing concentrations of exendin-4 (0.03-300 nM) were added to the organ bath cumulatively using a 2–6 min dosing schedule. KCl (120 mM) was added again to check the viability and contractility of the tissues. In some experiments, gut tissues (circular antrum, longitudinal jejunum, duodenum, ileum, and colon) were pre-contracted using carbachol (10 μM). After reaching a stable maximum contraction, increasing concentrations of exendin-4 (0.03-300 nM) were added cumulatively using a 2–6 min dosing schedule. At the end of each experiment, carbachol was washed out followed by adding KCl (120 mM) to check the viability of the tissues. A separate set of experiments examined the effect of exendin-4 on tissues contracted by electrical field stimulation (EFS). Briefly, after equilibration, gut tissues contracted by EFS to produce a sub-maximal contraction. The EFS parameters were: train duration, 5 sec; voltage, 50 V; pulse width, 0.5 ms; frequency, 10 Hz; interval, 1 min. After reaching a stable contraction, exendin-4 (300 nM) or atropine (1 μM) was added. At the end of each experiment, EFS was stopped and KCl (120 mM) was added to check the viability of the gut tissues. The volume of drug solutions added to the organ bath was less than 0.3% of the total bath volume.

### Blood glucose tolerance test

Twelve ferrets were fasted overnight and then anaesthetised with pentobarbitone (40 mg/kg, i.p.) to a depth sufficient to abolish the pedal reflex. Blood samples were collected by tail vein puncture at 5 to 20 min intervals and animals were randomized using Latin square design into four treatment groups (n = 3 per group). Saline (0.5 ml/kg) or exendin (9–39) (300 nmol/kg) was administered subcutaneously (25G, scruff of the neck). Fifteen minutes later, saline (0.5 ml/kg, s.c.) or exendin-4 (30 nmol/kg, s.c.) was injected, followed immediately by a glucose load (1.5 g/kg, i.p.). Blood sampling was continued for up to 2 h but as only one drop of blood was required for the glucose analysis saline replacement was not considered necessary. Blood glucose was measured immediately via a glucose oxidase-based method using a Glucometer Elite blood glucose meter (Bayer, Mishawaka, USA). Animals were killed at the end of the experiment with and overdose of pentobarbitone (80 mg/kg, i.p.).

### Implantation of radiotelemetric devices

Thirteen ferrets were fasted overnight but allowed free access to water. They were then injected with buprenorphine (0.05 mg/kg, s.c. Temgesic®), and anaesthesia was induced by ketamine (20 mg/kg i.m.; Alfasan, Holland) and maintained with 1.5% isoflurane (Halocarbon Products Corporation, USA) in O_2_ using a custom-made face mask and an anaesthetic machine (Narkomed 2C, Drager, USA). Animals were placed on a heating pad (UCI#390 Analogue moist heating pad, Rebirth Medical & Design, Inc., Taiwan) and the level of anaesthesia was assessed and monitored throughout the surgery by the pedal withdrawal reflex. Following a midline abdominal incision, a 19G needle was used to pierce the aorta, and then the catheter of a C50-PXT transmitter (Data Sciences, Inc, USA) was inserted up to a length of approximately 2 cm. A 2 × 2 mm piece of sterile gauze was placed over the catheter’s entry point, and fixed with a drop of tissue glue. The body of the transmitter was then sutured to the left side of the ferret’s abdominal wall muscle with the biopotential wires and catheter facing caudally. The gastric antrum was exposed and the biopotential wires were inserted into the muscle and secured in place by suturing the serosa. The abdominal cavity was sutured in closed layers and covered with a permeable spray dressing (Opsite®, Smith and Nephew, UK). Marbofloxacin (Marbocyl®, 2 mg/kg, s.c.) was administered once per day for 3 days and buprenorphine (0.05 mg/kg, s.c.) was given 8–12 h after the first dose. Animals were allowed to recover for seven days before drug treatment.

### Effects of food intake on gastric myoelectric activity and other physiological parameters

Four ferrets implanted with telemetry transmitters were used in a crossover design to assess the effect of food intake. Ferrets were fasted for 5 h, then at t = 5 h, animals were either presented with a food bowl containing 20 g of food (challenge A), or with an empty food bowl and remained under fasting conditions for a further 2 h (challenge B). Animals were allowed to recover for 3 days between the two challenges, the order of the challenges were randomised for each animal using a coin flipping strategy. At the end of experiment, animals were killed by an overdose of pentobarbitone (80 mg/kg, i.p.).

### Effects of exendin-4 (100 nmol/kg, s.c.) on emesis, food intake, gastric myoelectric activity, core temperature and the cardiovascular system

Nine ferrets were fasted overnight, and at 8:00 am they were presented with 20 g of food for 30 min. At 8:30 am, ferrets were fasted again. At 10:00 am (t = 0), they were either injected with saline (0.5 ml/kg, s.c.) or exendin-4 (100 nmol/kg, s.c.) in a crossover design with a seven day interval, the order of the challenges were randomised for each animal using a coin flipping strategy. At 2:00 pm (t = 4 h), ferrets were given 20 g of food. The amount of food eaten from 8:00 am to 8:30 am and from 2:00 pm to 3:00 pm was measured. At 3:00 pm, ferrets had free access to food until the end of the experiment (10 am the next day). During the experiment, physiological data were continuously acquired and emesis data were assessed for 4 h after drug injection.

### Data analysis

Prior to any statistical comparisons, the normality of the data was assessed with a Kolmogorov-Smirnov test. In the blood glucose tolerance test, the total area under the curve (AUC) for glucose was calculated using the trapezoidal rule and the significance of differences between treatments were assessed using one-way ANOVA followed by Bonfferoni multiple comparison tests. All telemetric data were processed using Spike2 (Version 7, Cambridge Electronic Design). The method for telemetric GMA data analysis had been described in previous studies; dominant power (DP) was defined as the highest power in the 0 to 15 cpm range, and dominant frequency (DF) was defined as the frequency bin with the highest power in the 0 to 15 cpm range [25]. Systolic blood pressure (BP) was calculated from the peak of the blood pressure recording trace and diastolic BP was calculated from the trough of the BP recording trace. Mean arterial BP was defined as Systolic BP/3 + 2*Diastolic BP/3 [31]. For heart rate (HR), the peak to peak interval was first calculated, and HR = 60/P-P interval (bpm). The standard deviation of N-N intervals (SDNN) was measured from the P-P wave interval of the BP in five minute segments and is used as an index of HRV [32]. Core body temperature (CBT) data were calculated by taking the average of the data. All the data were averaged in 1 h segments for statistical analysis. The differences of mean arterial BP, HR, HRV, CBT and GMA between saline and exendin-4 treatment groups and between food challenges were assessed using repeated two-way ANOVA and Bonferroni tests. The responses of the gut tissues were measured by the difference in the change in tension (g) before and after the addition of drugs. Responses were normalised and expressed as a percentage of the reference response produced by KCl. The significance of differences between data was determined using two-way ANOVA followed by Bonferroni tests. All data are expressed as mean ± s.e.m. Differences were considered statistically significant when *P* < 0.05. All statistical analyses were performed using GraphPad Prism version 5 (GraphPad Prism version 5.0, Inc. Version California, USA).

### Drug formulation

Exendin-4 and exendin (9–39) amide (American Peptide Company, Sunnyvale, CA) were dissolved in saline (0.9% w/v). Glucose (Merck, Darmstadt, Germany) was dissolved in saline (0.9% w/v NaCl, 154 mM) and administered in a volume of 0.5 ml/kg, i.p. Atropine methyl nitrate and carbachol chloride (Sigma, Saint Louis, USA) were dissolved in saline (0.9% w/v).

## Results

### Distribution of GLP-1 receptors

**Figure 1:**
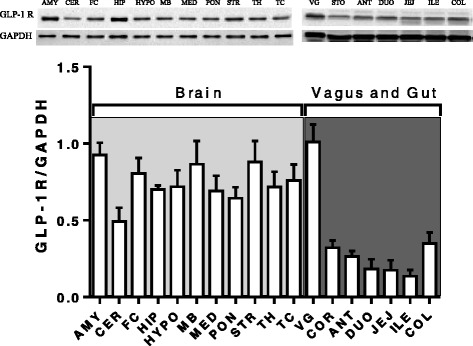
**The distribution of GLP-1 receptors in ferret brain and gastrointestinal tissues.** AMY, amygdala; CER, cerebellum; FC, frontal cortex; HIP, hippocampus; HYPO, hypothalamus; MB, midbrain; MED, medulla; PON, pons; STR, striatum; TH, thalamus; TC, temporal cortex; VG, cervical vagus nerve; COR, gastric corpus (body); ANT, gastric antrum; DUO, duodenum; JEJ, jejunum; ILE, ileum; COL, colon. Data represents the mean ± s.e.m. of 3 animals.

GLP-1 receptors were present in all regions of the brain and the gastrointestinal tract that were examined. Within the brain there was little variability in receptor distribution but in the gastrointestinal tract there was a clear regional differences in distribution with the highest density in the colon and the lowest in the ileum (Figure 1). Overall the density of GLP-1 receptors was approximately twice as high in the central nervous system compared to the gastrointestinal tract; these differences were not statistically significant (*P* > 0.05; n = 3, Figure 1).

### Effect of exendin-4 on the isolated gut tissues

**Figure 2:**
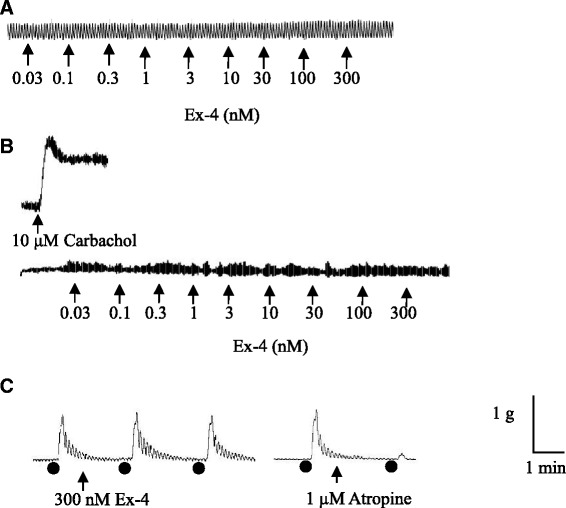
**Representative tracings illustrating the contractile responsiveness of ferret ileum to exendin-4, carbachol, electrical field stimulation (EFS) and atropine. (A)** Effect of exendin-4. **(B)** Effect of exendin-4 on the carbachol pre-contracted ferret ileum. **(C)** Effect of exendin-4 and atropine on the EFS pre-contracted ferret ileum. Ex-4 = exendin-4. Each black dot represents an EFS event.

The gut tissues exhibited regular spontaneous contractions, and the frequency and amplitude are shown in Table 1. KCl (120 mM) induced a rapid and reversible contraction in all gut regions, with the maximal response being observed within 5 sec (see Table 1). Exendin-4 (0.03-300 nM) failed to either contract any region of the gut or modify the amplitude and frequency of the spontaneous contractions (n = 3 for each region). In further experiments, therefore, carbachol (10 μM) was used to induce a contraction of the ileum to investigate if exendin-4 had a potential to induce relaxation of tissues. Carbachol produced a sustained contraction (see Table 1) but exendin-4 (0.03-300 nM) failed to affect the carbachol-induced contraction (n = 3). A final set of experiments examined the effect of exendin-4 on electrical field stimulated (EFS) contractions of the gut. The amplitude of EFS-induced contractions is shown in Table 1, but exendin-4 (100 nM) was without effect on the EFS-induced sub-maximal amplitude contractions (*P* > 0.05; n = 3). Conversely, atropine (1 μM) significantly reduced the amplitude of the EFS-induced contractions by 73.2% ±9.0%, 71.9% ±10.0% , 64.4% ±9.7% , 52.0% ±5.1% , 77.5% ±3.6% in the isolated segments of antrum, duodenum, jejunum, ileum and colon (n = 3), respectively (*P* < 0.05; n = 3). Representative tracings from the ileum are shown in Figure 2.

**Table 1:** **Summary of the contraction frequency and amplitude of the gastrointestinal tract tissues** ***in vitro***

	**Frequency of spontaneous contractions (cycles/min)**	**Amplitude of spontaneous contractions (g)**	**Amplitude of KCl response (g)**	**Amplitude of carbachol response (g)**	**Amplitude of EFS-induced response (g)**
Antrum	8.65 ± 0.23	0.62 ± 0.14	5.46 ± 0.33	2.33 ± 0.18	2.48 ± 0.72
Duodenum	17.02 ± 0.20	0.70 ± 0.11	4.12 ± 0.55	1.77 ± 0.12	0.96 ± 0.30
Jejunum	20.01 ± 0.12	0.69 ± 0.21	5.01 ± 0.27	2.80 ± 0.21	0.99 ± 0.09
Ileum	18.25 ± 0.25	0.69 ± 0.21	4.28 ± 0.11	1.19 ± 0.17	0.65 ± 0.12
Colon	15.78 ± 0.36	0.88 ± 0.30	6.25 ± 0.61	4.37 ± 0.70	2.69 ± 0.91

Data represents the mean ± s.e.m. of 3 animals.

### Blood glucose tolerance test

**Figure 3:**
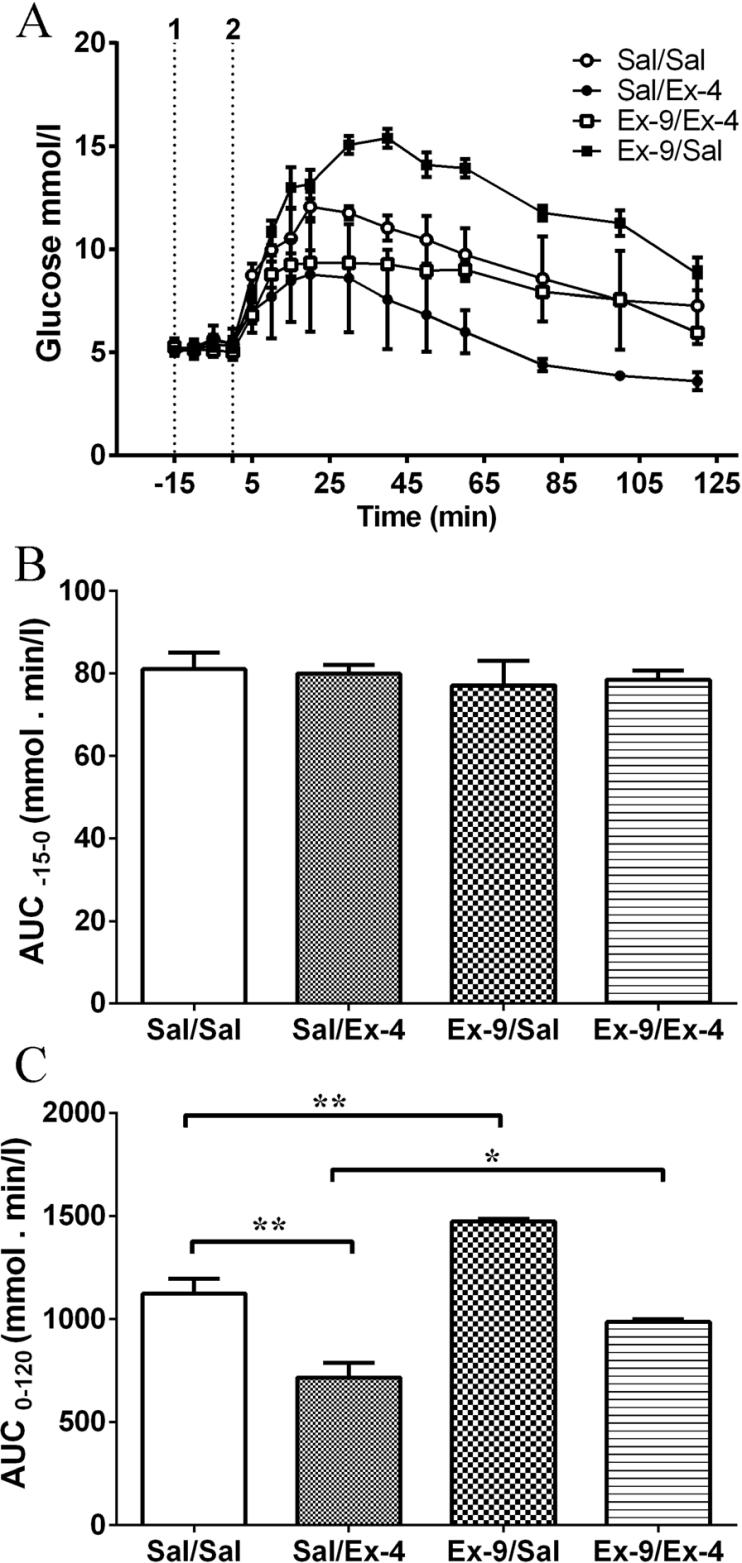
**Effect of a subcutaneous administration of exendin-4 (30 nmol/kg) and/or exendin (9–39) (300 nmol/kg) on blood glucose levels following an intraperitoneal glucose load (1.5 g/kg, i.p.). (A)** Blood glucose levels at t = −15 min to 120 min after administration of exendin (9–39) (300 nmol/kg) or saline (0.5 ml/kg) followed 15 min later by exendin-4 (30 nmol/kg) or saline (0.5 ml/kg) and a glucose load (1.5 g/kg, i.p.). **(B)** The area under the glycaemic curves between t = −15 and 0 min. **(C)** The area under the glycaemic curves between t =0 and 120 min. Sal = saline, Ex-4 = exendin-4, Ex-9 = exendin (9–39). 1. Ex-9 (300 nmol/kg, s.c.) or Sal (0.5 ml/kg, s.c.), 2. Ex-4 (30 nmol/kg, s.c.) or Sal (0.5 ml/kg, s.c.) + glucose (1.5 g/kg, i.p.). Data represents the mean ± s.e.m. of 3 animals. Significant differences are shown as *P <0.05, **P <0.01 (one-way ANOVA followed by Bonferroni test).

The basal blood glucose level was 5.1 ± 0.3 mmol/l (pooled data, n = 12; Figure 3A). The administration of exendin (9–39) or saline had no effect on blood glucose levels (AUC_-15 – 0_ values were not different between treatment groups, *P* > 0.05; Figure 3B). However, the administration of glucose (1.5 g/kg, i.p.) at t = 0 in the saline-treated animals caused a progressive elevation of blood glucose, which peaked at 20 min (max level was 12.1 ± 0.7 mmol/l, n = 3), and then decreased gradually thereafter (Figure 3A). In animals treated with exendin-4 (30 nmol/kg, s.c.), the effect was reduced; the AUC_0–120_ value was decreased by 36.6% (saline/saline 1124.0 ± 126.3 vs. saline/exendin-4 716.5 ± 123.0 mmol•min/l, *P* < 0.01; n = 3, Figure 3C). Administration of exendin (9–39) (300 nmol/kg, s.c.) 15 min prior to exendin-4 prevented the glucose lowering effect of exendin-4 (AUC_0–120_ values: saline/saline 1124.0 ± 126.3 vs. exendin (9–39)/exendin-4 986.9 ± 20.9 mmol•min/l, *P* > 0.05; n = 3, saline/exendin-4 716.5 ± 123.0 vs. exendin (9–39)/exendin-4 986.9 ± 20.9 mmol•min/l, *P* <0.05; n = 3, Figure 3C). However, administration of exendin (9–39) alone increased the blood glucose levels, with a significant increase being observed at 30 min after the glucose load (*P* < 0.05; Figure 3A). Thus, the AUC_0–120_ value was potentiated by 31.1% (saline/saline 1124.0 ± 126.3 vs. exendin (9–39)/saline 1473.0 ± 24.5 mmol•min/l, *P* <0.01; n = 3, Figure 3C).

### Effect of food intake on cardiovascular function, body temperature and gastric myoelectric activity

Telemetered ferrets have not been used extensively in studies on feeding, cardiovascular and gastrointestinal studies. Therefore, to provide a baseline for studies of exendin-4 we performed a preliminary study to investigate the effect of food intake itself on several physiological parameters in animals that had been fasted for 5 h.

**Figure 4:**
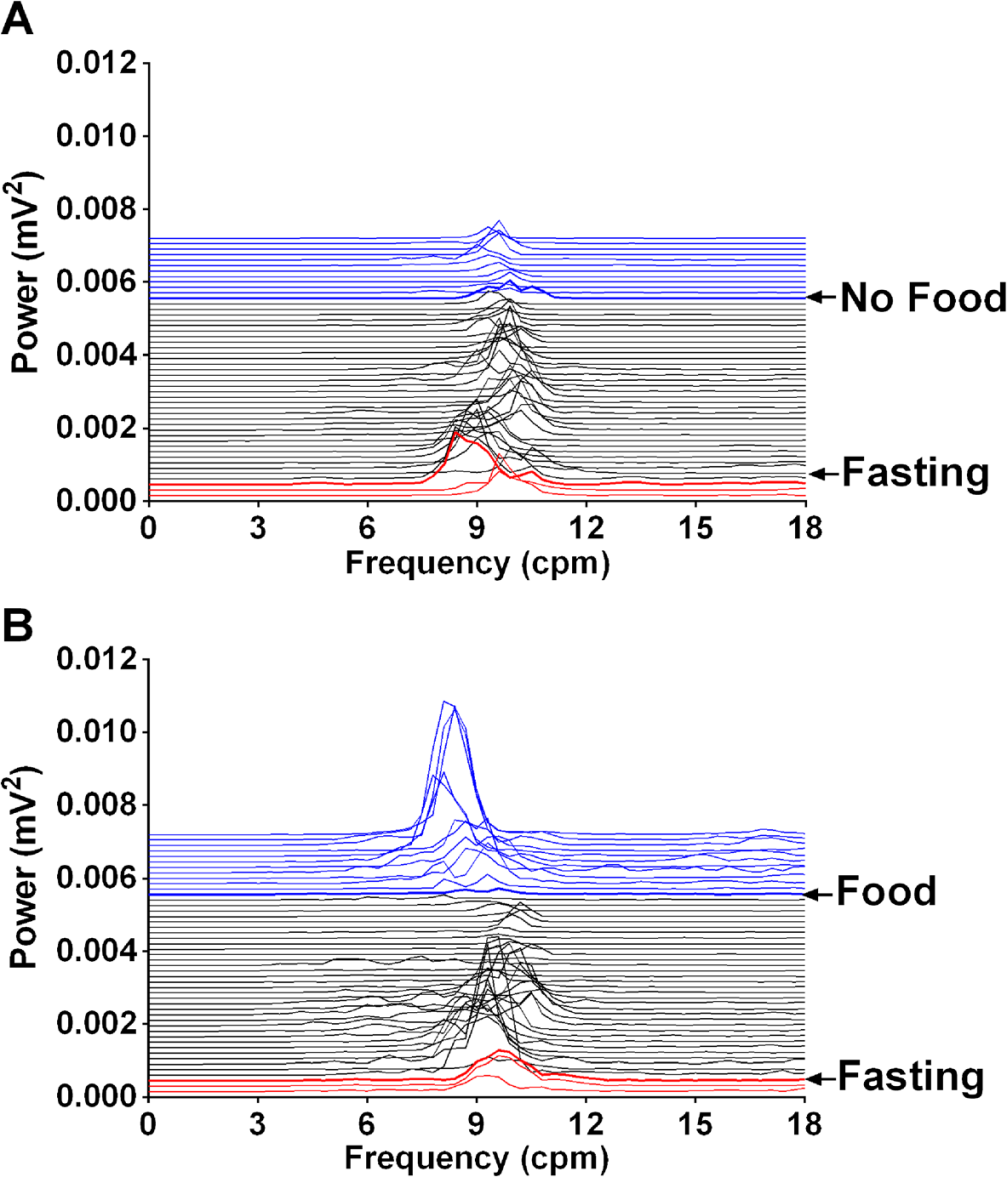
**Effects of feeding on gastric myoelectric activity. (A)** Running spectrum analysis of no food presented after fasting; **(B)** running spectrum analysis of food presented after fasting. Each horizontal line represents 10 min of data. The red lines represent the period of free feeding; the black lines represent the period of fasting; and the blue lines represent the period of food or no food.

**Figure 5:**
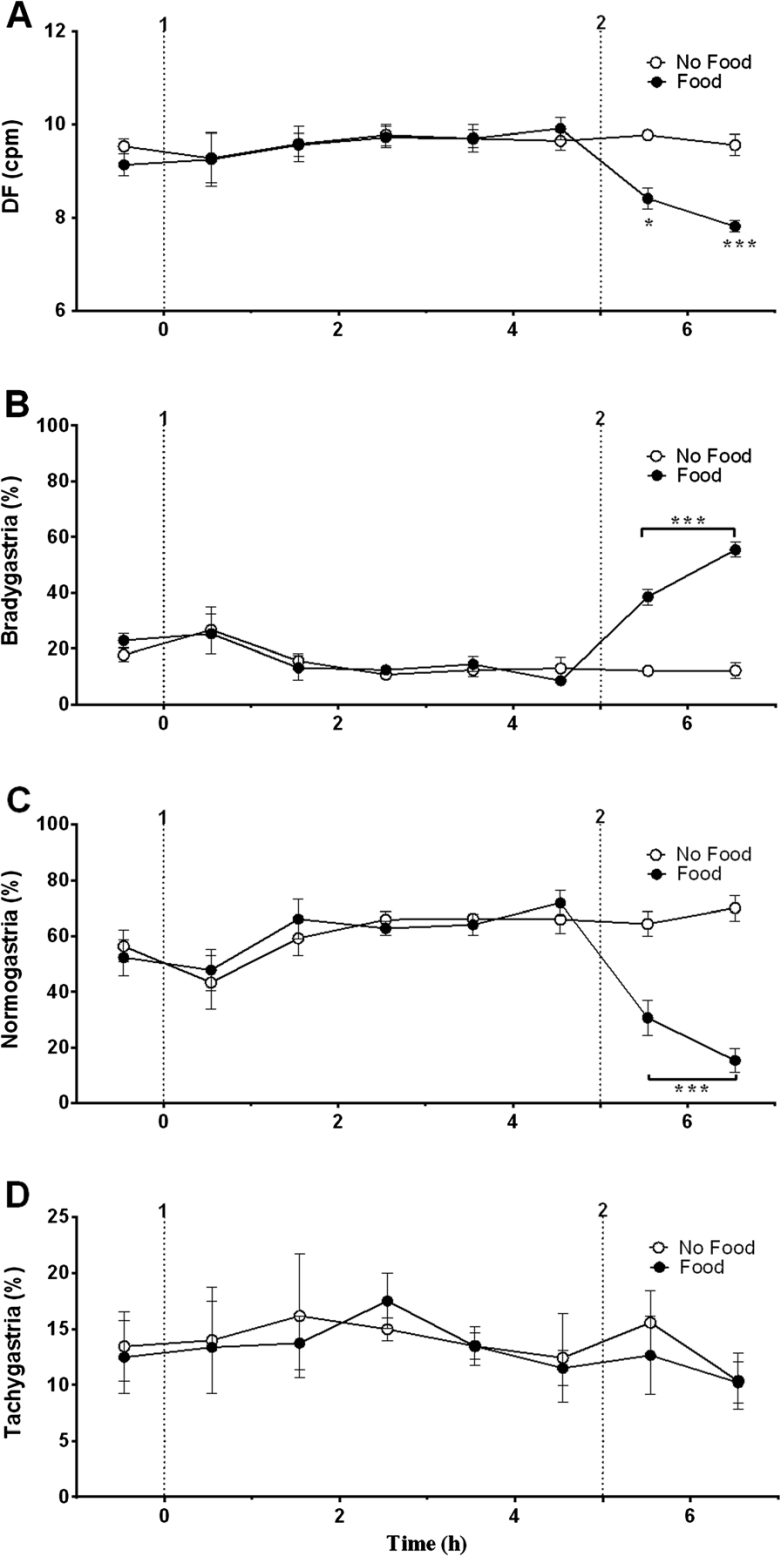
**Effect of food intake on gastric myoelectric activity. (A)** Dominant frequency, **(B)** bradygastria, **(C)** normogastria, and **(D)** tachygastria are shown. 1. Food withdraw in both group, 2. Food was presented to group A. Sal = saline, Ex-4 = exendin-4. Data represents the mean ± s.e.m. of 4 animals. Significant differences are shown as *P < 0.05, ***P < 0.001 (repeated measures two-way ANOVA followed by Bonferroni tests).

During fasting, ferrets had a basal mean arterial blood pressure of 128.5 ± 2.5 mmHg (systolic blood pressure 161.9 ± 1.8 mmHg; diastolic blood pressure 113.9 ± 0.9 mmHg) heart rate and heart rate variability was 238.6 ± 3.4 bpm and 0.079 ± 0.002 (arbitrary units), respectively (n = 3); core body temperature was 37.9 ± 0.1°C (n = 4, 2 replicates per animal). The baseline GMA recordings revealed a DF of 9.6 ± 0.1 cpm and a DP of 0.0019 ± 0.0006 mV^2^; 15.3 ± 2.8% of power was in the bradygastric range, 61.4 ± 4.1% of power was in the normogastric range, and 14.1 ± 0.7% of power was in the tachygastric range (n = 4, 2 replicates per animal). Feeding did not affect any of the cardiovascular parameters measured, or core body temperature (data not shown, n = 3). However, there was a decrease in DF to 8.9 ± 0.2 cpm, and a 30.3% increase of bradygastria, and a 41.2% decrease in normogastria in the animals that ate food (*P* < 0.001; n = 4, Figures 4 and 5).

### The emetic potential of subcutaneously administered exendin-4, and its ability to modify feeding, cardiovascular function, core body temperature, and gastric myoelectric activity

**Figure 6:**
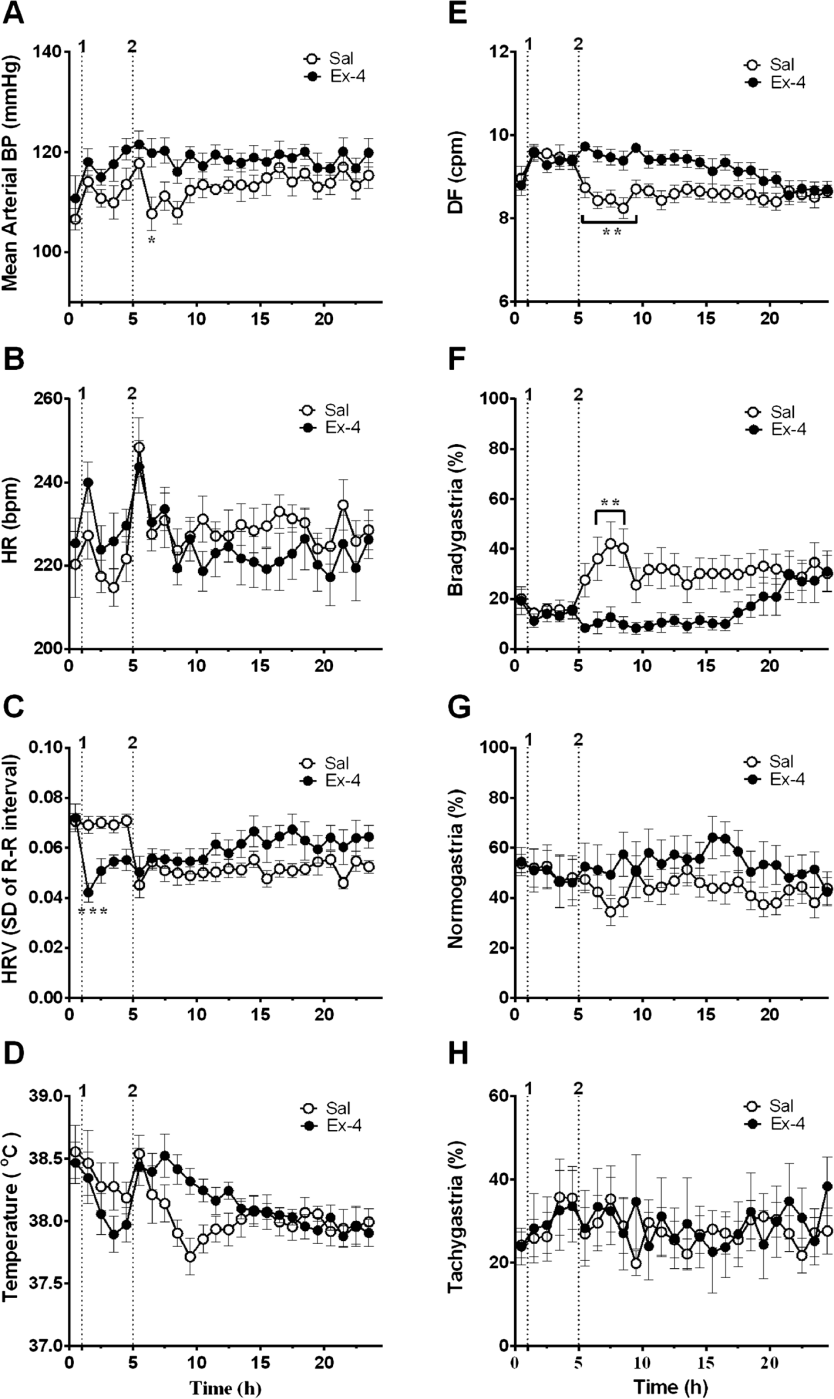
**Effect of a subcutaneous administration of exendin-4 (100 nmol/kg, s.c.) on cardiovascular functions, core body temperature and gastric myoelectric activity. (A)** Mean arterial BP, **(B)** heart rate, **(C)** heart rate variability, **(D)** core body temperature, **(E)** dominant frequency, power repartition in the **(F)** bradygastric, **(G)** normogastric and **(H)** tachygastric ranges are shown. 1. Injection of saline or exendin-4, 2. Food was presented to both groups. BP = blood pressure, HR = heart rate, HRV = heart rate variability, Sal = saline, Ex-4 = exendin-4. Data represents the mean ± s.e.m. of 8 animals. Significant differences are shown as **P < 0.01 (repeated measures two-way ANOVA followed by Bonferroni tests).

**Figure 7:**
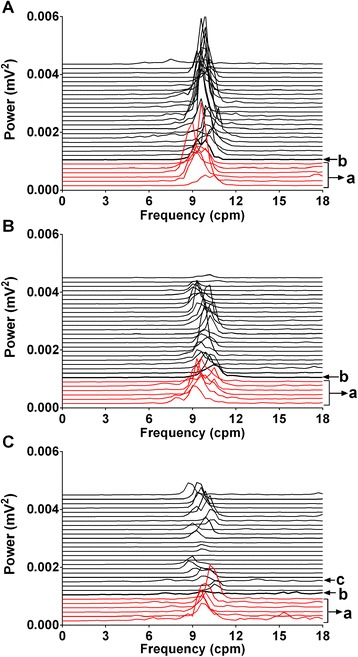
**Effects of a subcutaneous administration of exendin-4 (100 nmol/kg, s.c.) on gastric myoelectric activity. (A)** Running spectrum analysis of a control ferret that had received saline s.c.; **(B)** running spectrum analysis of a ferret that received exendin-4 but without emesis; **(C)** running spectrum analysis of a ferret that received exendin-4 inducing four episodes of emesis. a. baseline, b. saline or exendin-4 injection; c. during episodes of emesis. Each line represents 10 min of data.

Ferrets had a basal arterial blood pressure of 108.6 ± 2.5 mmHg (systolic blood pressure: 136.5 ± 3.2 mmHg; diastolic blood pressure: 92.3 ± 2.2 mmHg); heart rate and heart rate variability were 222.8 ± 4.9 bpm and 0.071 ± 0.003 (arbitrary units), respectively; core body temperature was 38.5 ± 0.1°C (n = 8, 2 replicates per animal). The baseline GMA recordings revealed a DF of 8.9 ± 0.2 cpm and a DP of 0.0015 ± 0.0002 mV^2^; 19.6 ± 3.2% of power was in the bradygastric range, 54.2 ± 3.3% of power was in the normogastric range, and 24.1 ± 2.6% of power was in the tachygastric range. The administration of saline did not affect significantly any of the cardiovascular parameters that were measured, or core body temperature (n = 8, Figure 6A-D) and did not induce emesis in any of the animals. However, the administration of exendin-4 (100 nmol/kg, s.c.) induced transient emesis (4 episodes of 26 retches and 4 vomits with a latency 36 min ) in one out of nine ferrets but decreased heart rate variability during the first hour following exendin-4 administration in all animals (*P* < 0.001; n =8, Figure 6C). In the ferret exhibiting emesis, there was an associated increase in mean arterial blood pressure and heart rate (approximately 30 bpm increase) with a corresponding trend for a decrease in body temperature (approximately 0.7°C fall, i.e. similar to that seen in animals without emesis); there was also a decrease in HRV (data not shown). Inspection of the electrogastrogram revealed that in the ferret exhibiting emesis, there was a shift towards bradygastria, and a reduction of power compared with animals that did not exhibit emesis following treatment with exendin-4 or saline (Figure 7). On presentation of food at t = 240 min, all of the saline-treated animals ate (8/8). Conversely, none of the animals (0/9) treated with exendin-4 ate. In the saline treated-animals that ate there was a consequent decrease of DF from approximately 9.8 cpm to 8.5 cpm with an increase in the % power of bradygastria from approximately 20% to 40% (*P* < 0.01; n = 8, Figure 6E and F).

## Discussion

The present studies are the first to demonstrate the ability of exendin-4 to lower blood glucose levels and inhibit feeding in the ferret. In the majority of animals, the effects occurred in the absence of a major action on cardiovascular or on gastric myoelectric activity (GMA). However, exendin-4 has a low emetic potential demonstrated by a single animal with brief retching and vomiting episodes. In this animal, there was a transient increase in BP and a disrupted GMA exemplified by a reduction of power and a shift towards bradygastria. This is in contrast to the bradygastria seen following eating, where there was a clear increase in power.

The activity of exendin-4 to lower blood glucose appears to be conserved across several species including diabetic mice [33], diabetic rats [34], *Suncus murinus* [19], healthy human subjects [35], and patients with type 2 diabetes [4]. In our studies, the specificity of the action of exendin-4 was confirmed using exendin (9–39), the GLP-1 receptor antagonist, which also elevated blood glucose levels alone suggesting that GLP-1 receptors are tonically active, and their activation lowers blood glucose. Indeed, exendin (9–39) is reported to correct fasting hypoglycaemia in SUR-1−/− mice, which are hypoglycaemic when fasted and hyperglycaemic when glucose-loaded [36]. Conversely, the role of GLP-1 receptors in control of gastro- intestinal motility is unclear and this may relate to species differences. There is no doubt in man that GLP-1 receptor activation *in vivo* inhibits gastric emptying [5] and small bowel motility [6], thereby reducing metabolic demand in association with food consumption and appetite [37-39]. In the mouse GLP-1 receptor activation relaxes carbachol-pre-contracted circular muscle strips of gastric antrum but not the fundus [40], while it inhibits the EFS-evoked circular contraction of the duodenum but not longitudinal contractions of isolated tissues [41]. Both effects are via an action on GLP-1 receptors in the enteric nervous system, probably via the release of nitric oxide [40]. In contrast in *Suncus murinus*, exendin-4 dose-dependently contracts isolated longitudinal ileum via the enteric nervous system and an involvement of muscarinic receptors [18]. Studies on isolated smooth muscle cells from the human colon show that GLP-1 (7–36) induces a weak contractile response that is abolished by exendin (9–39) amide [42]. However, GLP-1 failed to contract the circular antrum and corpus muscles in rats, but delayed gastric emptying via mechanisms probably involving the sympathetic system [16]. In the present studies, exendin-4 failed to either contract or relax isolated segments of the ileum (a similar lack of effect was also seen of antrum, duodenum, jejunum, colon tissues), even though GLP-1 receptors were detected in all areas of the GI tract that were investigated. The lack of response was unexpected given that we detected GLP-1 receptors in all regions of the gastrointestinal tract examined and the brain. Certainly, in other studies using similar methodology, GLP-1 receptors have been found in the pancreatic islets, lung, heart, gastrointestinal tract, and various brain areas, and GLP-1 receptor levels is highest within the phylogenetically oldest parts of the brain [43-46]. The specificity of the antibody for the ferret GLP1R receptor(s) is not known but the receptor is highly conserved with high inter- species (mouse, rat, dog, human) homology and the “ferret GLP1R” band appears in the same place (~53 kDa) as that seen in the rat brain using the same antibody (data not shown). However, it is recognised that further molecular characterisation of the putative ferret GLP1 R is needed before further studies of distribution and characterisation of pharmacology can be undertaken for comparison with other species.

More consistent data across species are seen from studies on GLP-1 receptors and feeding. GLP-1 receptor agonists reduce food intake across several species. In wild type mice, GLP-1 inhibits feeding following subcutaneous administration, but has no effect in GLP-1 receptor knockout mice [47]. In rats, peripherally administered-exendin-4 inhibits feeding at low doses via a vagal mechanism, but at higher doses the mechanism may also involve the nucleus tractus solitarius and hypothalamus [48]. In humans, GLP-1 is a potent regulator of food intake [49]. Therefore, the inhibitory action of exendin-4 on feeding in ferrets was not unexpected, and is also similar to data we obtained in *Suncus murinus* [20]. How exendin-4 inhibits feeding was not revealed in the present studies, but the mechanism does not appear to involve modulation of gastric myoelectric activity. This is particularly interesting, since studies have shown that GLP-1 agonists delay gastric emptying via vagal afferent inhibitory effects on gastric motor function in rats [50,51] and further studies have shown that GLP-1 and exendin-4-induced gastric motility inhibition is via activation of vagal non-adrenergic non-cholinergic (NANC) pathways and withdrawal of cholinergic tone [52].

The incidence of nausea and emesis in man following subcutaneously administered-exendin-4 at doses lowering elevated blood glucose is 43.5% and 12.8%, respectively [10]. It is interesting that the incidence of emesis (retching and vomiting) in the ferret (11%) is similar to that in man. This finding while interesting should be treated with a degree of caution as although the limitations of the ferret as a model for the assessment of anti-emetic agents particularly in ant-cancer chemotherapy are well described [22] data on translation of emetic liability of agents to humans is not as extensive [21] particularly in the case of peptides (e.g. CCK-8) [21]. Whilst it questionable whether it is possible to assess directly the effect of a drug to induce nausea in animals [24,53], activation of GLP-1 receptors induces conditioned taste aversion [54] and pica [55] in rats which lack an emetic reflex [14,15]. Food refusal can be a symptom of nausea in humans [56] and reduced food intake is a frequent occurrence in rats following administration of emetic agents [24] including exendin-4 [55]. Exendin-4 suppressed food intake in the ferrets in the present study but we are unable to determine if this is a primary effect on appetite or is secondary to induction of a sensation with a function equivalent to nausea in humans. However, in the ferret with an emetic response, we observed a concurrent decrease in the dominant frequency and a reduction in the power of slow waves in GMA that was not seen in the exendin-4-treated animals not exhibiting emesis. It is possible, therefore, that the decrease in the power of the bradygastric range is related to the mechanism of emesis, or that emesis *per se*, disrupted GMA. Although changes in GMA are often associated with nausea in humans in cases where nausea is mild such changes are not always observed [57] so the lack of effect of exendin-4 on GMA seen in the majority of animals does not preclude a “nausea –like” sensation being involved in genesis of the reduced appetite.

The subcutaneous injection of exendin-4 did not have an effect on mean arterial BP, HR, or core temperature. However, in our studies, SDNN is used as an index to represent HRV, and peripherally-administered exendin-4 decreased HRV during the first hour after drug injection reflecting a reduction in cardiac vagal tone. A decrease in HRV associated with nausea has been reported in several studies of motion sickness in humans [26,58,59] but in others an either an increase [60] or no change [61] has been reported with the variations possibly reflecting different stimuli and methods of analysis [27]. A decrease in HRV from an initial increase was associated with the onset of nausea in patients receiving anti-cancer chemotherapy [62].

The present study did not directly investigate the mechanisms involved in the effects of exendin-4 on food intake, HRV or emesis but a direct effect on gastrointestinal motility appears unlikely based upon the lack of effect in the *in vitro* studies. However, these observations do not preclude an effect on gastrointestinal motility mediated by vagal efferents either a consequence of a central action of exendin-4 (see below) or as a reflex consequence of exendin-4 action on abdominal vagal afferents [51]. Rodent studies support a central site of action of GLP-1 receptor agonists and sites implicated in GLP-1 receptor mediated effects on conditioned taste aversion [63], pica [55] and reduction in food intake [55,63] have all been shown in the present study to have GLP-1 receptors in the ferret. A central site of action of systemically administered exendin-4 is consistent with previous studies using *Suncus murinus* as exendin-4 is more potent to induce emesis when administered i.c.v. than it is following s.c. administration [20]. We propose that systemically administered exendin-4 acts on the area postrema to induce emesis but in contrast to agents with a high emetic potential (e.g. apomorphine, loperamide, cisplatin) [25,64], the low incidence of emesis will make this impossible to test using lesion techniques without the use of unacceptably large numbers of animals. The area postrema via connexions with the nucleus tractus solitarius could also be the site at which exendin-4 acts to reduce food intake and a decrease in HRV [24]. Interestingly, there is evidence in the ferret that the NTS itself has GLP-1 positive neurones [65]. However, as GLP-1 has been shown to activate abdominal vagal afferents in the rat an action on vagal afferents projecting to the area postrema/nucleus tractus solitarius can not be excluded.

## Conclusions

Our studies have revealed that peripherally administered exendin-4 reduces elevated blood glucose and inhibits feeding and has a low emetic potential in the ferret in the absence of major effects on blood pressure, or temperature homeostasis. A consistent decrease in HRV was identified. When emesis occurred, there was an increase in bradygastria and a reduction of power. It is proposed that the effects of exendin-4 on food intake, HRV and emesis occur by an action on the central nervous system and most likely the area postrema but this requires direct investigation. Identification of the site(s) at which GLP-1 receptor agonists act to produce these and the related dose–limiting toxicity of nausea will facilitate the development of agonists with only the desirable effects on glucose homeostasis.

## Abbreviations

BP: Blood pressure
CBT: Core body temperature
DF: Dominant frequency
DP: Dominant power
EFS: Electrical field stimulation
GLP-1: Glucagon-like peptide-1
GMA: Gastric myoelectric activity
HR: Heart rate
HRV: Heart rate variability
SDNN: Standard deviation of the N-N intervals
